# Integrative Analysis of Breast Cancer Cells Reveals an Epithelial-Mesenchymal Transition Role in Adaptation to Acidic Microenvironment

**DOI:** 10.3389/fonc.2020.00304

**Published:** 2020-03-10

**Authors:** Mehdi Sadeghi, Bryce Ordway, Ilyia Rafiei, Punit Borad, Bin Fang, John L. Koomen, Chaomei Zhang, Sean Yoder, Joseph Johnson, Mehdi Damaghi

**Affiliations:** ^1^Department of Cell and Molecular Biology, Faculty of Science, Semnan University, Semnan, Iran; ^2^Department of Cancer Physiology, Moffitt Cancer Center and Research Institute, Tampa, FL, United States; ^3^Proteomics Core, Moffitt Cancer Center and Research Institute, Tampa, FL, United States; ^4^Molecular Biology Core, Moffitt Cancer Center and Research Institute, Tampa, FL, United States; ^5^Microscopy Core, Moffitt Cancer Center and Research Institute, Tampa, FL, United States; ^6^Department of Oncologic Sciences, Morsani College of Medicine, University of South Florida, Tampa, FL, United States

**Keywords:** acid adaptation, EMT, tumor microenvironment, breast cancer, S100 family proteins

## Abstract

Early ducts of breast tumors are unequivocally acidic. High rates of glycolysis combined with poor perfusion lead to a congestion of acidic metabolites in the tumor microenvironment, and pre-malignant cells must adapt to this acidosis to thrive. Adaptation to acidosis selects cancer cells that can thrive in harsh conditions and are capable of outgrowing the normal or non-adapted neighbors. This selection is usually accompanied by phenotypic change. Epithelial mesenchymal transition (EMT) is one of the most important switches correlated to malignant tumor cell phenotype and has been shown to be induced by tumor acidosis. New evidence shows that the EMT switch is not a binary system and occurs on a spectrum of transition states. During confirmation of the EMT phenotype, our results demonstrated a partial EMT phenotype in our acid-adapted cell population. Using RNA sequencing and network analysis we found 10 dysregulated network motifs in acid-adapted breast cancer cells playing a role in EMT. Our further integrative analysis of RNA sequencing and SILAC proteomics resulted in recognition of S100B and S100A6 proteins at both the RNA and protein level. Higher expression of S100B and S100A6 was validated *in vitro* by Immunocytochemistry. We further validated our finding both *in vitro* and in patients' samples by IHC analysis of Tissue Microarray (TMA). Correlation analysis of S100A6 and LAMP2b as marker of acidosis in each patient from Moffitt TMA approved the acid related role of S100A6 in breast cancer patients. Also, DCIS patients with higher expression of S100A6 showed lower survival compared to lower expression. We propose essential roles of acid adaptation in cancer cells EMT process through S100 proteins such as S100A6 that can be used as therapeutic strategy targeting both acid-adapted and malignant phenotypes.

## Introduction

The principle driver of evolutionary processes is the concept of survival of the fittest. Those given populations that are the most well adapted to survive in an environment are the ones that will persist. In higher order organisms, the surviving populations are those that have a set of static traits that make them successful in a given environment. At a cellular selection level, organisms have the ability to acclimate to a given environment and alter their phenotype to be more successful in surviving. This ability to alter phenotype in order to acclimate to a given environment is particularly important in the context of cancer cell survival. In order for a cancerous cell population to persist, it must be able to adapt and evolve to maintain its' fitness within a given tumoral environment (1–3). Those cellular populations with the ability to more rapidly and efficiently adapt to the environment will have an advantage over the other cell populations when facing the challenges of a new or changing environment (4). Epithelial to mesenchymal transition (EMT) is one of the phenotypic switches that promote cancer progression, invasion and metastasis. EMT tests a cancer cells ability to efficiently change cellular states in response to changing conditions, also denoted as cellular plasticity, which also often referred to in the cancer stem cell model (5, 6). Although denoted as a transition, It has been recently observed that the EMT process is non-binary and occurs on a spectrum of transition states that can have the characteristics of both epithelial and mesenchymal phenotypes (7, 8). The transition to one of the intermediate states between epithelial and mesenchymal phenotype has been denoted partial EMT (pEMT), with cells expressing both markers of epithelial and mesenchymal cell status. pEMT states compared to complete EMT carry different migratory patterns during cancer metastasis (9, 10), and demonstrate the elevated plasticity of their epithelial progenitors (8). Another cause of EMT can be functional heterogeneity of cancer cells that is the result of genetic and epigenetic makeup as well as their interactions with the microenvironment. It has been recently shown that phenotypic heterogeneity is a dynamic reversible state of highly plastic cancer cells and their response to microenvironmental changes in GBM (11). Lately, there have been proposals for a strong connection between tumor plasticity and recreating intra-tumoral phenotypic heterogeneity (12) and also emphasizing the role of microenvironment in shaping spatial and temporal heterogeneity (13). It looks like the relationship between tumor cell plasticity, and intra-tumoral heterogeneity with emerging new phenotypes such as EMT or pEMT in everchanging cancer microenvironments is getting more attention and will be new area of research. It has been shown that growth factors, such as epidermal growth factor (EGF), transforming growth factor-β (TGF-β), and basic fibroblast growth factor (bFGF/FGF2) are also able to induce EMT (14, 15). It has also recently reported that tumor microenvironment conditions such as hypoxia and acidosis can induce EMT (16, 17).

Adenocarcinomas initiate and evolve within the hostile microenvironment of avascular ducts, which are characterized by acidosis, hypoxia, reactive oxygen species (ROS), and nutrient deprivation (18, 19). In particular, the acidic microenvironment of tumors strongly influences cancer progression and evolution. We have proposed that chronic acidosis induces genomic instability and selects for emergence of aggressive clones, leading to genomic diversity and increased tumor heterogeneity (20–24), a proximal cause of malignancy and resistance (25). Specifically, the acidified habitat imparts a Darwinian selection pressure that favors cells that adapt mechanisms to resist acid-mediated cell death. Further, the acidic microenvironment is also manifested in locally invasive cancers where it confers cancer cells a selective advantage over the stromal cells, leading them to invade to surrounding stroma. Indeed, an acidic microenvironment stimulates invasion and metastasis and also promotes remodeling of the extracellular matrix (ECM) (26–30). Further, acidosis promotes angiogenesis via the release of VEGF (31) and impairs immune surveillance (32, 33). Acid adaptation also pushes cancer cells toward a more aggressive phenotype through lysosomal redistribution (34) and plays a major role in subpopulation formation and evolution of solid tumors.

Integrative analysis has received a lot of attention lately in biology and cancer biology specifically, due to its nature of inter-validating data in different levels of biology such as genome, transcriptome, proteome, and metabolome (35). Different data integration approaches can help to combine various high throughput omics data to construct an integrative regulatory network. These networks can help to understand the molecular basis of carcinogenesis and provide a powerful framework for exploring new cancer biomarkers (36, 37). With the advancements in network inference and construction methods, network analysis, and interpretation approaches it is feasible now to explore authentic and accurate molecular signatures. Another advantage of such analysis is discovery of groups of co-regulated molecules as a sub-network biomarker for treatment, diagnosis or prognosis applications.

Expression profiling is a major key to unraveling gene expression patterns and the transcriptome. RNA sequencing is a next-generation sequencing (NGS) technology that sequences cDNA in order to provide accurate measurement of transcripts levels to define biological networks (38). Networks are the language of complex systems like biological systems. Biological networks are used widely to model biological interactions at the molecular level to understand biological processes particularly in the case of cancer (39). To assess biological networks different techniques have been developed; centrality analysis is one of them (40, 41). Centrality analysis ranks the nodes (genes in gene regulatory networks) based on their significance. In centrality analysis, adding topological parameters to biological data leads to sufficiently informative results that have been shown to be effective in exploring key signature molecules in biological processes (42). Such biological network analysis has been used in cancer biomarker discovery (43).

Here in, we studied the effect of acid adaptation on early stage breast cancer evolution using the MCF7 cancer cell line. We studied EMT phenotypic switches as regulators of acid adaptation using RNA sequencing data and gene regulatory network analysis and by integrating the results to SILAC proteomics data. For that reason, we compared acid-adapted MCF7 breast cancer cell line RNA profile to parental MCF7 cells. The differentially expressed genes in the acid-adapted cells were used to construct a gene regulatory network. This network was implemented to explore sub-network biomarkers related to EMT by a set of robust criteria. We then compared our findings with the SILAC proteomics results and found S100 family proteins such as S100A6 and S100B are abundant in both sets of omics data. We validated both S100B from RNA sequencing and S100A6 from proteomics data, by Immunocytochemistry (ICC). We further our validation using IHC of breast cancer patient TMAs with 160 biopsy cores. S100A6 expression was compared to LAMP2b as a biomarker of acidosis in solid tumors, and each core's LAMP2b expression was co-registered with S100A6 expression using Definiens tissue studio software analysis. The TMA co-registration analysis showed correlation of S100A6 with LAMP2b expression the most in early breast cancer stage, ductal carcinoma *in situ*, DCIS. Survival analysis of patients with different expression of S100A6 revealed correlation of high S100A6 expression with worse outcome in survival of breast cancer patients. When taken in total, we conclude that amongst many paths of EMT, S100 proteins play critical roles in acid-induced EMT that can be responsible for cancer progression and survival of cancer cells in their continuously changing microenvironments.

## Materials and Methods

### Cell Culture and Acid Adaptation *in vitro*

MCF7 cells were acquired from American Type Culture Collection (ATCC, Manassas, VA, 2007–2010) and were grown in DMEM-F12 (Life Technologies) with 10% fetal bovine serum (HyClone Laboratories) and 1% peniciline/stroptomycine added. Growth medium was buffered with 25 mmol l^−1^ each of PIPES and HEPES and the pH adjusted to 7.4 or 6.5. Cells were tested for mycoplasma contamination and authenticated using short tandem repeat DNA typing according to ATCC's. To achieve acid adaptation, cells were chronically cultured and passaged directly in pH 6.5 medium for ~2 months. Chronic low-pH-adapted cells underwent at least 20 passages.

### RNA Sequencing

RNA sequencing was performed on MCF7 and acid-adapted MCF7 cells using the NuGen Ovation Encore Complete RNAseq kit, which generates strand-specific total RNAseq libraries (Nugen, Inc., San Carlos, CA). Following quality control screening on the NanoDrop to assess 260/230 and 260/280 ratios, the samples were screened on the Agilent BioAnalyzer RNA Nano chip to generate an RNA Integrity Number (RIN) (Agilent Technologies, Santa Clara, CA). Hundred nanogram of DNase-treated total RNA was then used to generate double-stranded cDNA, which was initiated with selective random priming allowing for the sequencing of total RNA, while avoiding rRNA and mitochondrial transcripts. After primer annealing at 65°C for 5 min, a first strand cDNA synthesis reaction was performed at 40°C for 30 min using kit-supplied reverse transcription reagents. Second strand cDNA synthesis was performed in a 70 μl reaction volume at 16°C for 1 h and the reaction was stopped by adding 45 μl of stop solution. The double-stranded cDNA was then fragmented to ~200 bp with the Covaris M220 sonicator (Covaris, Inc., Woburn, MA), followed by purification with Agencourt RNAClean XP (Beckman Coulter Life Sciences, Indianapolis, IN). The fragmented DNA was suspended in 10 μl of water and end repair was performed in a 13 μl for 30 min at 25°C, followed by a heat inactivation of 70°C for 10 min. Sample-specific indexed adapter was ligated to the end-repaired DNA for 30 min at 25°C, followed by a two-step strand selection process with an intervening 1.8X volume RNAClean XP bead purification. 13 cycles of library amplification and a 1.2x volume RNAClean XP purification of the strand-selected library was performed, followed by resuspension of the library DNA in 30 μl of RNase-free water. Final libraries were screened for library fragment size distribution using an Agilent BioAnalyzer High sensitive DNA Chip. Libraries were then quantitated using the Kapa Library Quantification Kit (Roche Sequencing, Pleasanton, CA), normalized to 4 nM, and were sequenced on an Illumina NextSeq 500 150-cycle high-output flow cell in order to generate ~40 million paired-end reads of 75-base per sample (Illumina, Inc., San Diego, CA) (44).

### RNA Sequencing Data Processing and Analysis

The RNA-seq data analysis workflow has been provided schematically in Supplementary Figure 1. Raw reads were quality-filtered to obtain clear data via removal of adaptor sequences, ambiguous or low-quality reads and reads with more than 5% N, using FastQC version 0.11.8 (http://www.bioinformatics.babraham.ac.uk/projects/fastqc/) and Trimmomatic version 0.39) (45). Then clean reads were aligned to the reference genome (GRCh37) using HISAT2 version 2.1.0 (46). Finally, the read count values for aligned sequences of genes were computed to represent the expression levels of genes using HTSeq version 11.1 (47). Differentially expressed genes (DEGs) between two groups were explored using R (48) package DESeq2 version1.24.0 (49).

Genes with *p*-value <0.05 were selected as differentially expressed Genes. Benjamini-Hochberg (BH) multiple testing correction was applied on results.

## Proteomics

### SILAC Labeling

Acid-adapted and naive cells were labeled by SILAC. Cells were cultured in heavy SILAC media (Δ6-lysine and Δ10-arginine) for eight doubling time of MCF7. Extent of labeling was determined by LC–MS/MS analysis of tryptic peptides from labeled samples to ensure >90% labeling.

### Lysis and Digestion

Cells were lysed by sonication in a buffer of 50% trifluoroethanol and 50 mM ammonium bicarbonate, pH 8.0, and protein was measured by the Bradford method. Protein from heavy- and light-labeled cells was combined in equal amounts, and lysis buffer was added to bring the final volume to 200 μl. The combined protein was reduced with 100 μl of 40 mM TCEP/100 mM dithiothreitol for 1 h at 37°C. Proteins were alkylated with 100 μl of 200 mM iodoacetamide for 30 min in the dark at ambient temperature. The volume of the reduced and alkylated sample was brought to 1 ml with 50 mM ammonium bicarbonate, pH 8.0. Trypsin was added at a ratio of 1:50 and samples were digested at 37°C overnight. Digests were frozen at −80°C and lyophilized. Dried peptides were resuspended in HPLC water with 0.1% TFA and desalted on 100-mg Thermo hypersep C18 columns. Eluted peptides were dried in a Speed-Vac and resuspended in HPLC water for isoelectric focusing fractionation.

### Isoelectric Focusing Fractionation

Tryptic peptides were fractionated using a narrow-pH-range fractionation strategy. At the end of the isoelectric focusing programme, strips were manually cut into 20 fractions. Peptides were extracted and samples were combined in the following manner to achieve 15 fractions for LC–MS/MS analysis: (anode end) samples 1–2, 3–4, 5–6, 7–8, and 9–10 were combined to make five fractions, samples 11–20 were left as individual fractions.

### LC–MS/MS

Samples were analyzed as duplicate injections for each fraction. A nano-flow ultra-high performance liquid chromatograph (RSLC, Dionex, Sunnyvale, CA) coupled to an electrospray ion trap mass spectrometer (LTQ-Orbitrap, Thermo Scientific, San Jose, CA) was used for tandem MS peptide-sequencing experiments. The sample was first loaded onto a pre-column (2 cm × 75 μm ID packed with C18 reversed-phase resin, 5 μm particle size, 100 Å pore size) and washed for 8 min with aqueous 2% acetonitrile and 0.04% trifluoroacetic acid. The trapped peptides were eluted onto the analytical column (C18 Pepmap 100, 75 μm × 50 cm ID, Dionex). The 120-min gradient was programmed as: 95% solvent A (2% acetonitrile + 0.1% formic acid) for 8 min, solvent B (90% acetonitrile + 0.1% formic acid) from 5 to 15% in 5 min, 15 to 40% in 85 min, then solvent B from 50 to 90% B in 7 min and held at 90% for 5 min, followed by solvent B from 90 to 5% in 1 min and re-equilibration for 10 min. The flow rate on the analytical column was 300 nl min^−1^. Ten tandem mass spectra were collected in a data-dependent manner following each survey scan. The MS scans were performed in the Orbitrap to obtain accurate peptide mass measurements, and the MS/MS scans were performed in the linear ion trap using a 60-s exclusion for previously sampled peptide peaks. Mascot (www.matrixscience.com) searches were performed against the UniProt human database downloaded on 11 July 11 2012. Two missed tryptic cleavages were allowed, the precursor mass tolerance was 1.2 Da to accommodate selection of different isotopes of the peptide precursor. MS/MS mass tolerance was 0.6 Da. Dynamic modifications included carbamidomethylation (Cys), oxidation (Met), heavy lysine (Δ6) and heavy arginine (Δ10).

Quantification of differences in protein expression between SILAC-labeled samples was performed as described using MaxQuant. Results were filtered to require a posterior error probability (PEP) score < 0.05 and summed intensity > 0. Candidates were selected among proteins that consistently showed at least a 1.5-fold increase under low-pH conditions across label-flipping experiments.

#### Network Construction

The STRING database is a valuable resource for the exploration and analysis of functional gene/protein interactions (50). STRING database was used to find conserved experimentally validated gene-gene interaction networks for the explored DEGs. Since STRING builds protein-protein interaction (PPI) networks thereby our network was constructed upon coding RNAs.

#### Motif Exploring and Motif Ranking

Networks consist of smaller and repetitive structural units which are called motifs. Network motifs can be described as recurring circuits of interactions from which the networks are made (51). Motifs have important roles in biological networks and suggested that they accomplish overriding functions in biological networks. In this study, Cytoscape (52) NetMatchStar plugin (53) was used to find 3-node 3-edge network motifs in the gene regulatory network which retrieved from STRING database.

In order to further our network analysis, multiple topological and biological parameters were determined and used. Log2 fold change of differentially expressed genes associated in the gene regulatory network (Supplementary Table 1), association of network's genes with biological processes involved in EMT (based on explored GOBP terms related to EMT) (Supplementary Table 2) and gene prioritization score (Supplementary Table 3) which were obtained from Cytoscape GPEC (54) plugin (54), were considered as biological parameters. Betweenness centrality and node degree are two network topological parameters (Supplementary Table 4) which obtained using Cytoscape (52) NetworkAnalyzer (55) plugin and were considered besides biological parameters for network's robust motif ranking. Node degree indicates the number of connected edges to each node and betweenness centrality shows the control level of a node over interactions of other nodes in a network. This centrality parameter prefers the nodes that allow to connect non-directly connected clusters of a network.

The next step was to find the most important motifs in the network. For this purpose, a ranking scheme (56) was performed based on a multi objective weighting function. This scheme is based on parameters which we gathered before: (i) Topological parameters, node degree and betweenness centrality, (ii) the presence of motif genes in EMT related biological pathways (see “Discussion” for more detail), (iii) the gene prioritization score obtained from Cytoscape GPEC plugin (54), (iv) acid-adapted MCF7 cell lines gene expression log2 fold-changes (based on differential expression analysis of acid-adapted MCF7 cell lines vs. non-adapted cell lines). Using this weighted multi-objective function in Equation 1, the motif ranking was performed.

$$\begin{array}{l} GS_{ij}=\;\frac{w_{1j}}{2}.\frac{{\langle nD\rangle }_{i}}{max\left(nD\right)}+\frac{w_{1j}}{2}.\frac{{\langle nB\rangle }_{i}}{max\left(nB\right)}+w_{2j}.\frac{{\langle PP\rangle }_{i}}{max\left(PP\right)}\\ \;+w_{3j}.\frac{{\langle GPS\rangle }_{i}}{max\left(GPS\right)}+w{\;}_{4j}.\frac{{\langle \left|LFC\right|\rangle }_{i}}{max\left(LFC\right)} \end{array}$$

*GS*_*ij*_ is the ranking score for each motif (*i* = 1… *n*) in different weighting scheme (*j* = 1… 13) as said in Table 1. Different weighting values including w_1j_ to w_4j_ are used to strike importance of used factors, <nD>_i_: average node degree for motif's node, <nB>_i_: average betweenness centrality of each node in a motif, <PP>_i_: number of genes in a motif involved in EMT related pathways, <GPS>_i_: average gene prioritization score obtained from GPEC, <|LFC|>_i_: average absolute log2 fold change for each motif.

**Table 1:** Weighting scenarios for motif ranking.

**Sets**	**w_**1**_**	**w_**2**_**	**w_**3**_**	**w_**4**_**
Set 1	1	0	0	0
	0	0	1	0
	0	0	0	1
Set 2	1.4	0	0	3.4
	0	1.4	0	3.4
	0	0	1.4	3.4
Set 3	1.8	1.8	0	3.4
	1.8	0	1.8	3.4
	0	1.8	1.8	3.4
Set 4	1.16	1.16	1.8	3.4
	1.16	1.8	1.16	3.4
	1.8	1.16	1.16	3.4
Set 5	1.4	1.4	1.4	1.4

Five different sets of weighting scenarios including 13 different weighting schemes were applied (Table 1) to remove biasness between used parameters in motif prioritization. Each set pays more attention to specific parameters in Equation (1). In the first set, only one parameter is more important for ranking. In the sets 2–4, two, three and four parameters are important, respectively, and constantly have higher weights to the absolute LFC of the motif to explore phenotype-specific top ranked motifs. In the fifth set, equal weights allocated to all the parameters. This weighting scheme leads to 13 ranking score for each motif. After removing duplicated motifs, we selected the top 10 motifs from each weighting scenario for further analysis (Supplementary Table 5).

## Proteomics and Transcriptomics Integrative Data Analysis

Integrative proteomics and transcriptomics data analysis was performed in roder to ensure about consistency of proteomic and transcriptomic data regarding explored motifs. In this regard 19 differentially expressed genes of the top 10 explored motifs cross referenced with SILAC proteomics data (DCIS and MCF7 cell lines) to see which of the following transcriptomes are alternatively translated in the proteomics level.

## Examining Survival and Gene Alteration Changes

cBioportal.org was used to examine the survival and gene alteration changes in breast cancer patient samples. For non-invasive breast cancer sample data, the set from Razavi et al. (57) was used, and for invasive breast cancer sample data the set from Curtis et al. (58) was used.

## Immunofluorescence

Cells cultured at pH 6.5 chronically and pH 7.4 of with the same passage were rinsed with PBS, fixed in cold Methanol:Acetone (1:1) for 10 min and then blocked with 4% bovine serum albumin in PBS for 1 h. Samples were incubated with primary antibody of S100B and S100A6(1:100) and secondary Alexa-Fluor 488 antirabbit (1:500) antibody) for 1 h in room temperature. Coverslips were mounted using ProLong Gold Antifade Reagent (Life Technologies) and images were captured with a Leica TCS SP5 (Leica) confocal microscope.

## Immunohistochemistry

For human tissues, a TMA containing formalin-fixed and paraffin-embedded human breast tissue specimens was constructed in Moffitt Cancer Center histology core. The TMA contains 27 normal breast tissue, 30 DCIS, 48 invasive ductal carcinomas without metastasis, 49 invasive ductal carcinomas with metastasis and 48 lymph node macro-metastases of breast cancer. Cores were selected from viable tumor regions and did not contain necrosis. A 1:400 dilution of anti-LAMP2 (#ab18529, Abcam), anti-S100A6 antibody (Prestige Antibodies Powered by Atlas Antibodies, Sigma-Aldrich) and anti S100 protein were used as primary antibodies. Positive and negative controls were used. Normal placenta was used as a positive control for LAMP2, normal breast was used as a positive control for S100 and normal kidney was used as a positive control for S100A6. For the negative control, an adjacent section of the same tissue was stained without application of primary antibody, and any stain pattern observed was considered as non-specific binding of the secondary.

Immunohistochemical analysis was conducted using digitally scanning slides and scoring by three independent reviewers. The scoring method used by the pathologist reviewer to determine (1) the degree of positivity scored the positivity of each sample ranged from 0 to 3 and were derived from the product of staining intensity (0–3^+^). A zero score was considered negative, score 1 was weak positive, score 2 was moderate positive, and score 3 was strong positive (2). The percentage of positive tumors stained (on a scale of 0–3).

## Statistical Analysis

Statistical analysis and estimation of correlations in this study were performed using GraphPad Prism v.6. Correlation significance calculated by Pearson correlation. The *p*-values reported for survival analysis measured by cox regression hazard ratio and log rank tests. All paired tests were performed by Student's *t*-test.

## Results

### RNA Sequencing of Acid-Adapted and Non-adapted MCF7 Cells Unravels the EMT Mechanism of Breast Cancer Cells

**Figure 1:**
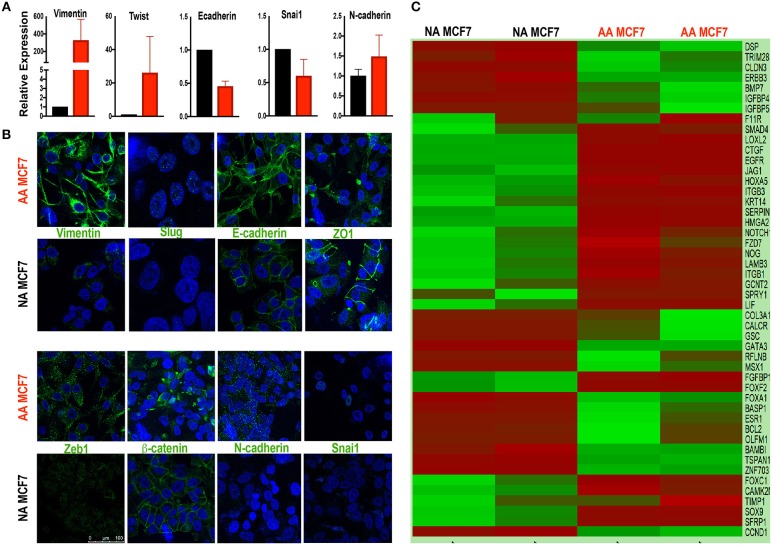
Acid adapted cells show partial EMT phenotype. **(A)** q-RT-PCR-analysis and **(B)** IF of EMT marker at RNA and protein level respectively show both markers of epithelial and mesenchymal phenotype are present in acid adapted cells confirming their transient EMT phenotype. **(C)** Analysis of RNA sequencing shows a mixed epithelial and mesenchymal markers. Heatmap plot for EMT related deferentially expressed genes in AA-MCF7 compared to MCF7. Each row represents a gene and each columns stands for a sample. Cells color is correlated to gene count in the corresponding sample. Color code for gene count: red, high expression; green, low expression.

In order to study the effects of acidosis on EMT of breast cancer cells at early stages such as ductal carcinoma *in situ* (DCIS) we first probed the effect of chronic acid adaptation on EMT status of MCF7 breast cancer cell line using quantitative reverse transcription-polymerase chain reaction (qRT-PCR) (Figure 1A) and Immunofluorescent (IF) (Figure 1B) techniques. Acid adaptation showed some of the epithelial to mesenchymal phenotypes such as high expression of Vimentin or loss of membrane β-catenin and ZO-1 and didn't show some other's such as loss of E-Cadherins (Figures 1A,B). So, we concluded acid adaptation is a path to complete EMT and the status we observed can be explained as partial EMT induced by acid adaptation that can be completed by further adaptation to acid or other microenvironmental conditions (Figures 1A,B). The partial EMT is reported in other publications and referred as a measure of plasticity (8, 10). Then we carried out sequencing of RNA on a paired sample of MCF7 cells and its acid-adapted counterpart. MCF7 cells are ER, PR, and HER2 positive with many phenotypes of early neoplastic cells such as slow metabolism, and low rate of glycolysis and Warburg phenotype that makes them a proper model of studying acidosis at early stages of breast cancer (27, 59). They are also tumorigenic but not metastatic i.e., injection of MCF7 into immunodeficient mice will result in tumor growth but not metastasis. For RNA extraction we used acid-adapted and non-adapted MCF7 (parental) at the same passage number with similar growth rate at the time of experiment. We identified 1,928 differentially expressed genes in acid-adapted MCF7 cells compared to non-adapted MCF7 (Supplementary Table 1). Using STRING database, a regulatory interaction network based on experimentally validated interactions was plotted. The constructed network was replotted in Cytoscape software for better visualization (Supplementary Figure 2). Then we searched for EMT related markers in the RNA sequencing data and found that acid adapted cells show some of epithelial markers and some of the mesenchymal markers validating the partial EMT statues of acid adapted cells (Figure 1C).

#### Gene Regulatory Network

**Figure 2:**
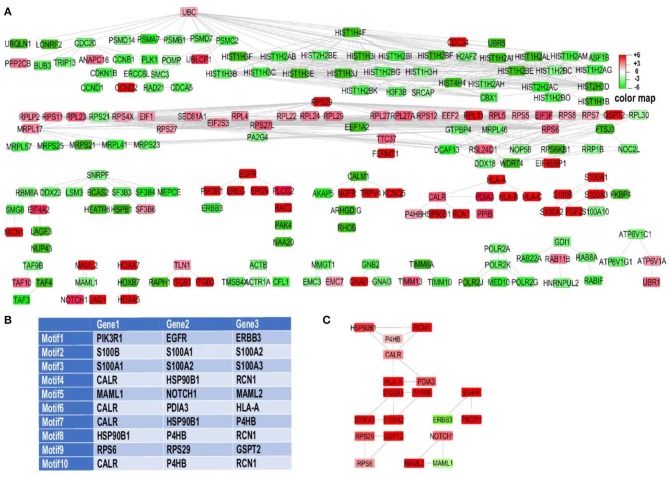
RNA sequencing motif analysis unravels EMT related genes involved in acid adaptation. **(A)** Experimentally validated gene regulatory networks of differentially expressed genes. For better visualization Y files layout algorithm of cytoscape was used to organize the network. Two node interactions and disconnected nodes were ommited. **(B)** Top ten ranked motifs of our network, directed toward EMT. **(C)** Top 10 explored motifs based on ranking analysis were merged together. The association of some of genes like P4HB and CALR in multiple motifs which present in top 10 motifs leads to construct a small sub-network by merging of these motifs which leads to construct core regulatory subnetwork.

To obtain an interaction network, an effort to unravel the regulatory core related to EMT under the influence of acidosis was made through identifying and ranking 3-node and 3-edge motifs (Figure 2A). To this end, *n* = 3,320 three member motifs were identified in the network using Cytoscape NetMatchStar plugin. In order to take the significance of motifs in cellular EMT into account, GOBO terms related to EMT were explored. Then for motif ranking scheme a factor was considered for each motif based on the membership of its genes in these terms. In order to place more emphasis on EMT Cytoscape GPEC plugin was used for gene prioritization based on explored GOBP terms. It works based on a random walk with restart algorithm. GPEC helps to rank genes based on their association with specific diseases or biological pathways (EMT in our case) The obtained scores were considered as another weight in scoring function (60). The log fold change, node degree and betweenness centrality were used in the scoring function as well. Using these factors in the scoring function the explored motifs were prioritized and ranked. The top 10 ranking motifs (Figure 2B) were selected for enrichment analysis toward EMT and acid adaptation. These motifs consist of 19 unique genes. Merging of these top ranked motifs leads to construct the underlying core subnetwork of the genes that were affected by acidosis and are related to EMT, differentiation and invasion of the tumor cells (Figure 2C).

### Integrative Analysis of Transcriptomics and Proteomics of Acid-Adapted and Non-adapted MCF7 Cells Reveals the Role of S100 Proteins in Acid-Induced Epithelial to Mesenchymal Transition

**Figure 3:**
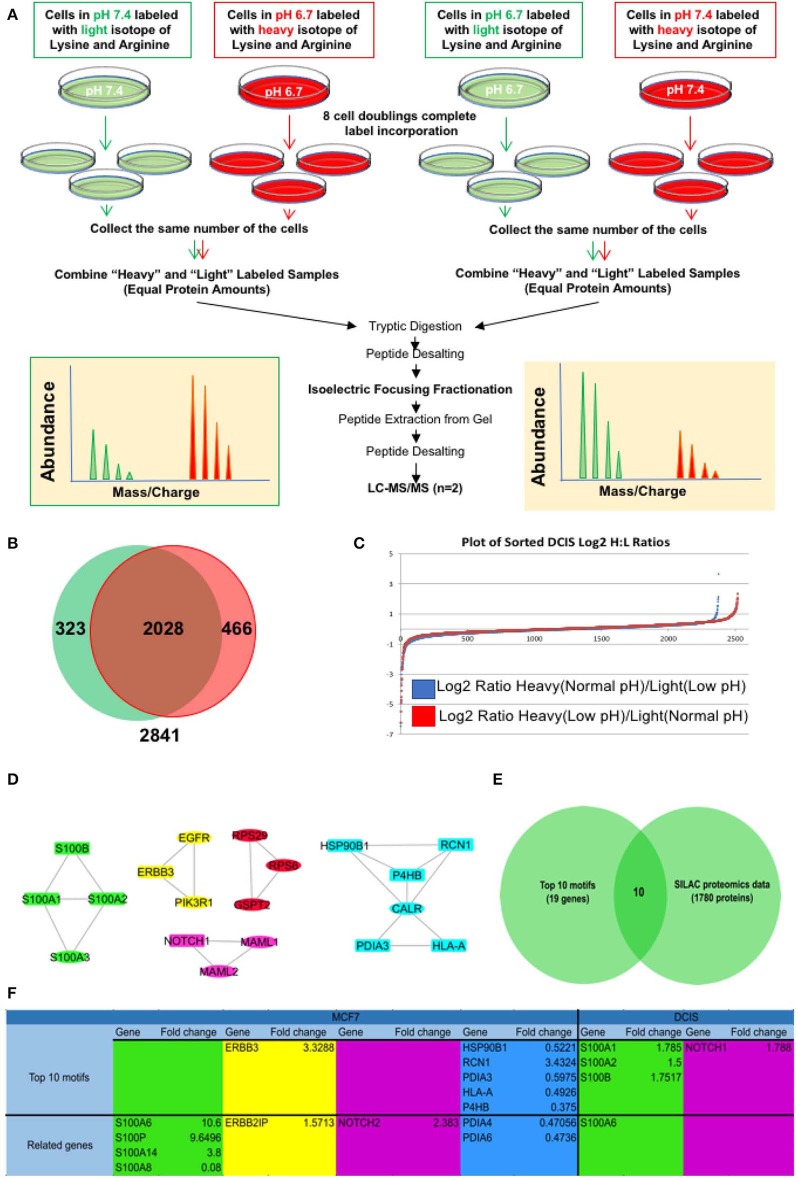
Integrative analysis of proteomics and transcriptomics data to discover the acidic microenvironment induced EMT genes. **(A)** A schematic of our SILAC proteomics design. We flipped the labeling to make sure the changes in protein expression is not affected by the type of labels. **(B)** Venn diagram and **(C)** Log 2 fold change of SILAC proteomics data discovered in each flipping experiment. **(D)** Integrated interaction map of the regulatory subnetwork and their related altered proteins in both DCIS and MCF7 cell lines. **(E)** Venn diagram indicating that among *n* = 45 transcripts (The subnetwork and it's near interactions) *n* = 12 proteins were differentially translated with the abundancy of S100 family. **(F)** The name of proteins that are discovered in DCIS and MCF7 proteomics and are correlated to the motif's from RNA sequencing data.

For further validation of our findings in RNA sequencing and EMT related motif analysis at the protein level, we compared all the genes in the EMT motifs with their relative protein change in our SILAC discovery proteomics of the MCF7 cell line published previously (27) as well as the MCF-DCIS (DCIS) cell line which we conducted SILAC proteomics on for this study. Since the focus of this study is on early adaption of breast cancer cells we selected DCIS cell lines and adapted them to acid for 3–6 months in the same process as the MCF7 cells. The SILAC proteomics approach was applied to compare the whole proteome of acid-adapted cancer cells to non-adapted counterparts. SILAC or stable isotope-labeled amino acids in cell culture is a quantitative mass spectrometry (MS) based technique that is used to compare the proteome of pairs of biological samples (61) which in our case is acid-adapted and acid-naive breast cancer cell lines. To minimize the rate of false-positive biomarker association, parallel SILAC experiments were conducted for each cell lines in which the acid-adapted or non-adapted cells were labeled by growing them in SILAC “heavy” media (^13^C_6_ lysine and ^13^${\text{C}}_{6}^{14}$N_4_ arginine), while the comparator cells (acid-naive or acid-adapted cells, respectively) were cultured in media containing the corresponding amino acids of naturally occurring isotopic distribution. The labeling strategy was reversed (flipped) to eliminate potential bias due to the media and incorporation of the stable isotope-labeled amino acids (Figure 3A) (62). MCF7 data was previously published for biomarker discovery of acid adaptation (27). In DCIS SILAC proteomics, 2,841 proteins were detected with 466 unique proteins for acid-adapted DCIS cells and 323 unique proteins for non-adapted ones (Figures 3B,C and Supplementary Figure 3). We used fold change to plot our data and used 1.5-fold change cut off (Figure 3C). The same analysis and cut off was applied for both DCIS and MCF7 cells. To do integrative analysis, we looked for any proteins related to the five explored motif packs isolated from RNA sequencing data (Figure 2C) in both MCF7 and DCIS proteomics with more than 1.5 ratio change in acid-adapted vs. non-adapted condition (Figure 3C). In order to perform integrative proteomics and transcriptomics data analysis we focused on 10 explored motifs based on motif ranking analysis (Figure 2C). This analysis has been conducted to ensure consistency of proteomics and transcriptomics data. Translational pattern of 19 differentially expressed genes were assessed in MCF7 and DCIS proteomics data. We plotted the interactome map for these altered proteins that were identified through integration of transcriptome and proteome data (Figure 3D). In this figure nodes in rectangular shape have both gene expression and protein translation alteration and oval nodes only present alterations in transcriptomics level. Ten proteins out of 19 discovered genes had more than 1.5-fold change in MCF7 and DCIS proteomics data (Figure 3E). Among these genes the ones presented in Figure 3F are differentially expressed at the proteomics level in the DCIS and MCF7 cell lines (Figure 3F). Due to abundancy of the S100 family proteins in both transcriptomics and proteomics data, this motif pack was chosen for further experimental validation.

### Acid-Adapted MCF7 Cells Express Higher S100A6 and S100B Proteins

**Figure 4:**
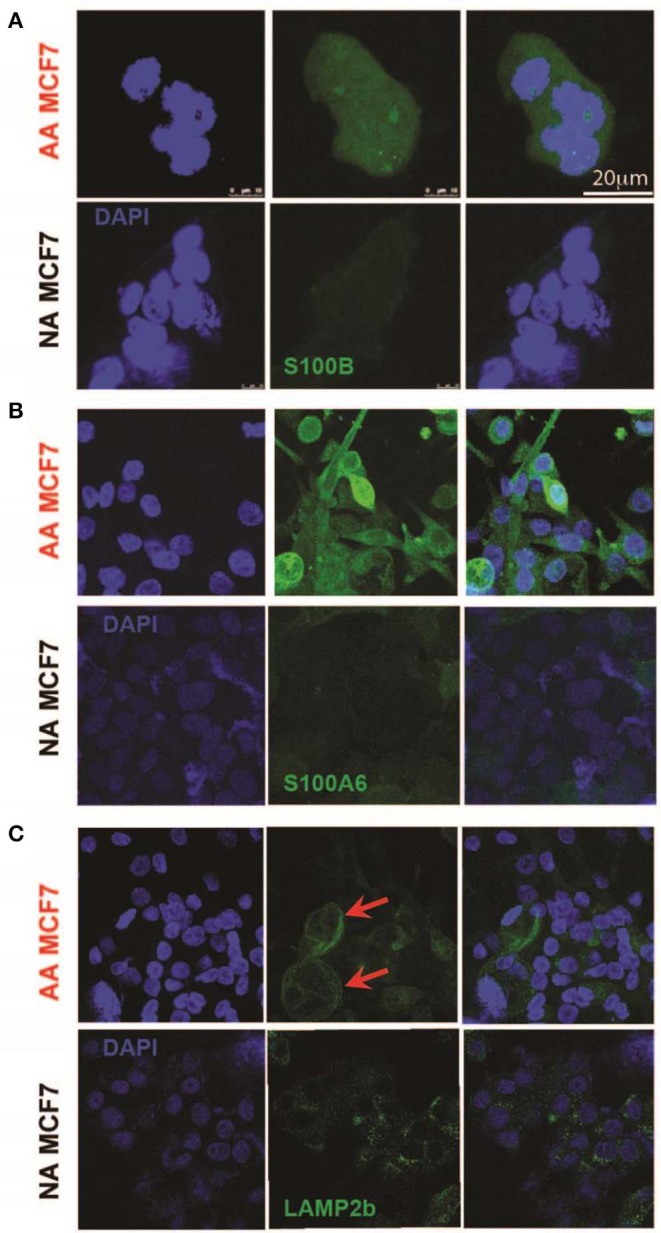
Validation of higher expression of acid-induced EMT markers by Immunocytochemistry. **(A)** S100B protein expression in acid-adapted and non-adapted MCF7 cells with the analysis on right. S100B expression is significantly higher in acid adapted cells. **(B)** S100A6 ICC of acid-adapted and non-adapted MCF7 cancer cells shows higher expression of S100A6 in AA MCF7 cells. **(C)** LAMP2b ICC of acid-adapted and WT MCF7 cancer cells. Acid-adapted MCF7 cells display membrane localization of LAMP2b, compared to cytoplasmic localization in non-adapted MCF7 cells.

To further validate the S100 motif discovered in both RNA sequencing and proteomics data in acid-adapted EMT analysis, we performed Immunocytochemistry (ICC) experiments on our acid-adapted and non-adapted MCF7 cells. We chose S100A6 and S100B from the family because of over expression of S100A6 at the protein level in both MCF7 and DCIS cells and S100B as one marker discovered in RNA sequencing of MCF7 cells and the proteomics of DCIS. To do the experiment, both AA MCF7 and NA MCF7 were seeded on the one slide with eight chambers on it and were treated with exactly equal amounts of antibodies. Slides were imaged using a Leica TCS SP5 confocal microscope with exact settings for both cells, and samples were imaged the same day. We found higher expression of both S100B and S1006 in acid-adapted MCF7 cells (Figures 4A,B). To confirm the acid adaptation status of our cells, we also stained the acid-adapted MCF7 cells and the non-adapted MCF7 cells with the known marker of acid adaptation, LAMP2b. We observed membrane localization of LAMP2b in our acid-adapted MCF7 cells (Figure 4C), which is characteristic of acid-adapted cell populations.

### S100A6 Expression Correlates With Survival in Breast Cancer Patients

**Figure 5:**
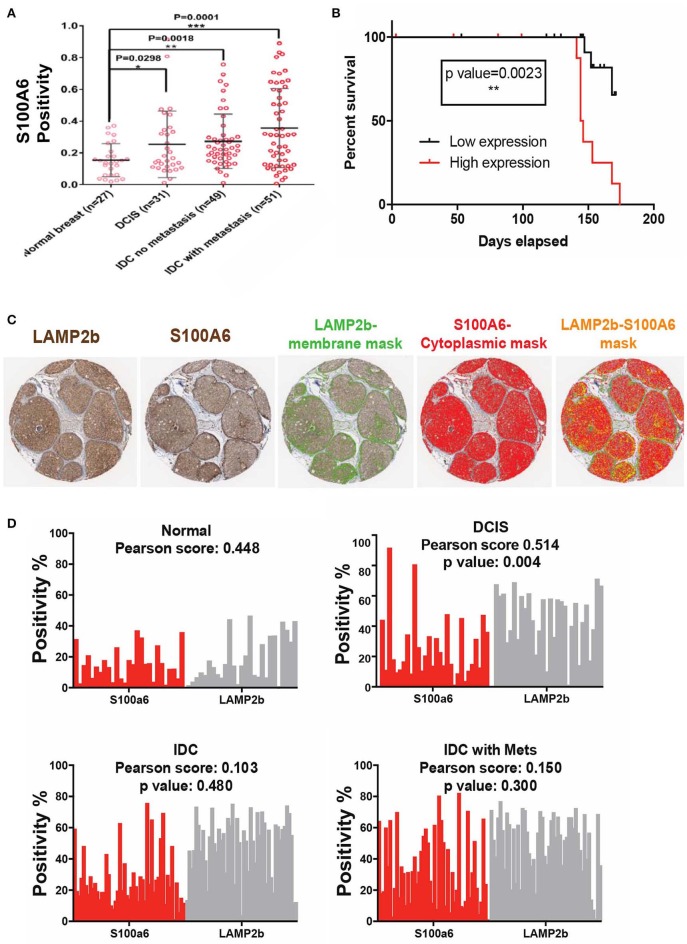
Clinical validation of S100A6 expression correlation to acid phenotype in breast cancer. **(A)** TMA analysis of 160 biopsy cores stained with S100A6 antibody showed increased expression of this protein from normal to DCIS, IDC, and IDC with Mets. Data are shown as mean with standard deviation as error bar. **(B)** Kaplan-Meier graph comparing DCIS patient's survival with low expression of S100A6 (Below the average) to patients with high S100A6 expression. Patients with high expression survived less than patients with low expression. **(C)** Representative images of core biopsies stained for both LAMP2b and S100A6 on sequential cuts. **(D)** Correlation analysis of LAMP2b and S100A6 in different stages of breast cancer.

We then sought to clinically validate our identified S100 proteins expression in breast cancer patient Tissue Micro Arrays (TMA) that we have available at the Moffitt Cancer Center tissue core bank. On the basis of our previous findings, we hypothesized that an acidity biomarker should have two characteristics. First, due to the increase in glycolytic rate with breast cancer progression, there should be an association of progression with marker of acidity and second, the expression of the proteins should correlate somehow with the expression pattern of LAMP2b as it is a known marker of acidosis (27, 34). In short, S100A6 and S100B proteins should increase with stage similar to LAMP2b. To test this, we analyzed protein expression of S100A6 and S100B via IHC of TMAs containing patient sample biopsies from different stages of breast cancer totaling 160 cores. While the protein expression of S100A6 showed statistically (*P* < 0.0001) higher in tumor samples compared with adjacent normal breast there was no difference for S100B. The negative results of S100B could be the cause of problems with antigen specificity or epitopes that were used. We then continued our analysis with S100A6 by measuring the positivity of each core in different stages of breast cancer. Increased S100A6 expression correlates with increased tumor progression from DCIS to invasive ductal carcinoma (Figure 5A). There were notably significant differences between normal breast and DCIS, Invasive Ductal Carcinomas (IDCs), and IDCs with local metastases indicating the role of this protein in cancer progression and invasiveness. We then compared the survival of patients with high and low expression of S100A6 for each biopsy cores in three categories of DCIS, IDC and IDC with local metastasis. For defining high vs. low expression, we use the median of all the cores in each category as middle point and anything below the media was taken as low and vice versa. The data was analyzed using two testing methods: Mantel-Cox and Gehan-Greslow-Willcoxon. The DCIS category showed significant difference between low and high expression (Figure 5B), which confirms our previous studies of DCIS as the most acidic tumors in breast cancer. The difference wasn't significant for survival of patients with breast cancer at IDC, and IDC with local invasion stages, implying the importance of acidosis and acid related phenotype at early stages of cancer again (Supplementary Figure 4).

To further prove the correlation of S100A6 and acidosis we compared the positivity of LAMP2b as a marker of acidosis and S100A6 as our candidate, for each biopsy core in our TMA. Comparative analysis of S100A6 positivity from each biopsy core to LAMP2b expression of the same core showed a correlation between these two proteins (Figures 5C,D) validating the role of S100A6 in acid adaptation.

## Discussion

Deregulated energetics is a hallmark of cancer progression, and the deregulation of cellular energetics has a profound effect on the growth and progression of a tumor. The creation of an acidic tumor microenvironment (TME) is one of these major consequences of deregulated cancer cell energetics. When faced with the acidic TME the cancer cell population must either adapt or perish, with the former being the usual outcome due to the extraordinary ability of cancerous cell populations to adapt to a changing environment. This adaptation to an acidic TME is not a passive action and leads to permanent changes in the phenotype of the surviving population. Little is known about the phenotypic changes that occur throughout the arduous task of adapting to the acidic TME, and deeper insight into these changes will move us a step in the direction of targeting these aggressive populations therapeutically.

Although the concept of lower pH in the tumor microenvironment is not a new discovery, the specific studying of acid-adapted cancer cell phenotypic switch is a relatively new realm of science. Previous investigations have found numerous phenotypic changes that occur during cancer cell populations adapting to an acidic environment such as, chronic autophagy (63), increased presence of lysosomal proteins in the plasma membrane (27), and heightened aggressiveness (34). Acidity in the intratumoral environment, not associated with acid adaptation, has also been shown to foster the stemness of cancer cell populations in osteosarcoma (64).

The aim of this study was to understand the role of acidic microenvironment in the EMT phenotypic switch, a demonstration of cancer plasticity and heterogeneity of cancer cell populations, and study their role in patient survival. We used a unique approach to identify vital regulatory sub-networks that are involved in the acid adaptation of cancer cell populations using integrative analysis of transcriptomic and proteomic data of selected cancer cells under an acid microenvironment that mimics one of the harsh selection pressures amongst many in solid breast tumors. The advantage of our approach is that our network analysis workflow encompasses different layers of information such as log fold change in cells, involvement of genes in partial and complete EMT processes and network centrality parameters which reflects gene regulatory role in the whole network. These considerations led to isolation of the motifs that have a critical role in cancer cells' acid adaptation and pEMT. The discovered motifs also have significant regulatory function throughout the network from a structural perspective. Network centrality parameters were considered as a unique factor to weighting nodes. Log fold changes of motif genes were another parameter to rank motifs. Therefore, we have four parameters to rank the motifs: direct association of motif in EMT, motif prioritization score which is based on Cytoscape GPEC plugin and reflect indirect association of motif in EMT, centrality of the gene within the network, and expression behavior of motif in acidosis. When taken in total, these four parameters return the important motifs within the system.

Here in, we demonstrated the correlation of cancer cells acid adaptation, EMT and its driven heterogeneity with patient's survival. Our findings demonstrated a partial EMT phenotype in our acid-adapted cellular populations by correlation to EMT markers accepted in the field. This partial transition may represent a heightened degree of plasticity or metastatic ability, with cells carrying phenotypic characteristics of both epithelial and mesenchymal cells. We observed downregulation of Snai1 in the acid-adapted group, which negatively correlates with E-cadherin expression, and is not typical of a traditional EMT switch. While this was not typical of the EMT response, we did observe EMT characteristics with heightened vimentin and N-cadherin expression. Due to the observed changes in EMT markers caused by acid adaptation, we believe the acid adaptation may target specific pathways in the EMT process, while neglecting others. We also proposed one of the possible mechanisms of acid-induced EMT phenotypic alteration through S100 family proteins, specifically S100A6 and S100B proteins. These findings can be used for therapeutic advances targeting EMT and heterogeneity of breast tumors while also providing a better understanding of the mechanism behind microenvironment induced phenotypic changes toward EMT, and the role EMT plays in acid-induced cancer progression and evolution.

## Data Availability Statement

The datasets generated for this study can be found in the NCBI Gene Expression Omnibus (GSE145383).

## Ethics Statement

All the patient data for breast TMA4 used in this study were deidentified by Moffitt Cancer Center and consent was obtained from the participants at the source. The authors of this manuscript had no access to patient information in any course of this study.

## Author Contributions

MD designed and accomplished the experiment and wrote the manuscript. MS and IR analyzed the data. BO, PB, BF, and CZ did the experiments. JJ analyzed the images. JK and SY supervised the proteomics and transcriptome analysis.

### Conflict of Interest

The authors declare that the research was conducted in the absence of any commercial or financial relationships that could be construed as a potential conflict of interest.
