# Supplementary material for: Integrative Analysis of Breast Cancer Cells Reveals an Epithelial-Mesenchymal Transition Role in Adaptation to Acidic Microenvironment
Source: Front Oncol. 2020 Mar 10;10:304. doi: 10.3389/fonc.2020.00304 (PMC7076123; doi:10.3389/fonc.2020.00304)
Supplement: Supplementary file 1 [file Data_Sheet_1.pdf]

Supplementary figure 1.

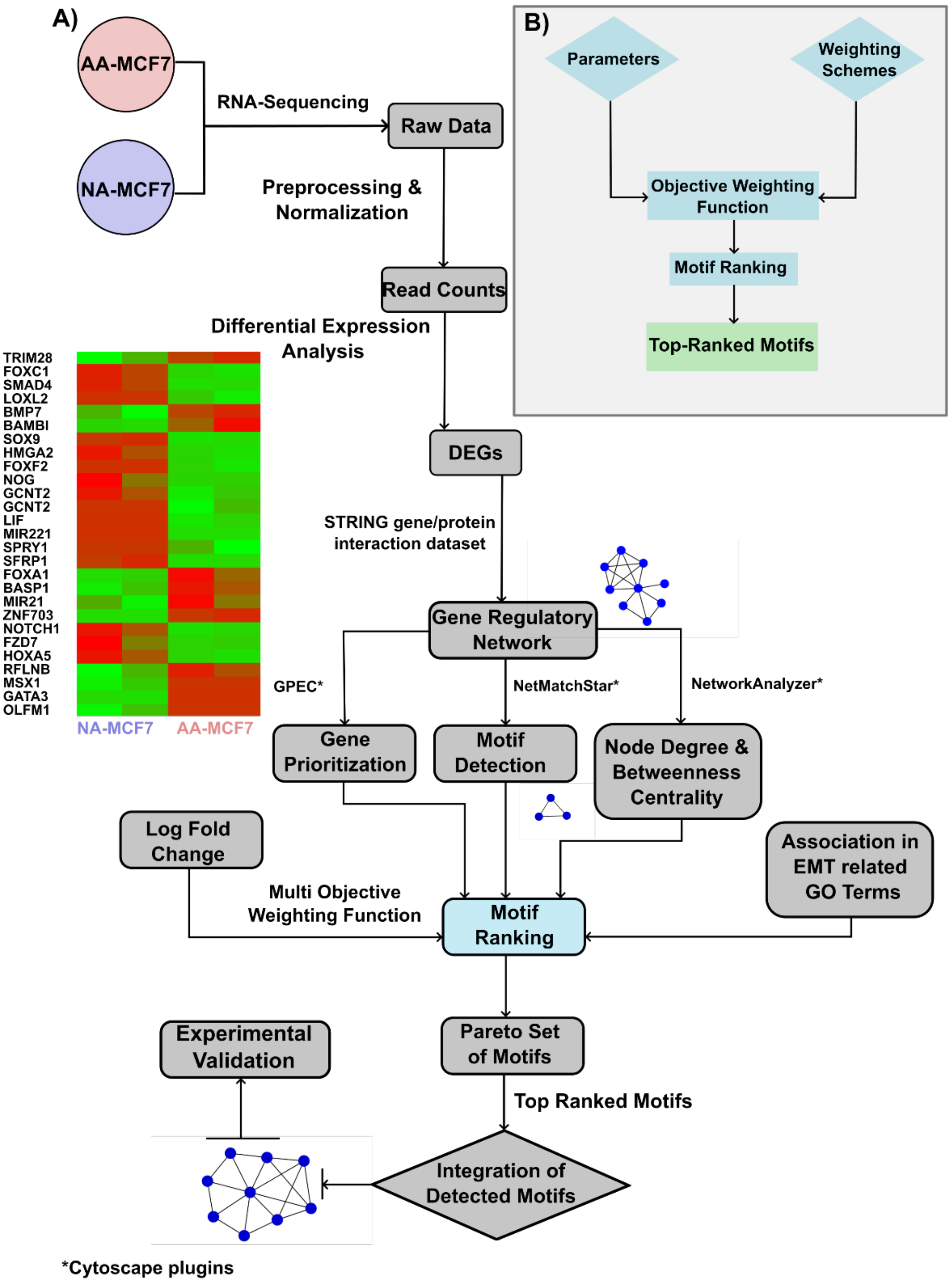

Sup figure 1: RNA sequencing data analysis workflow and the methodology of network analysis. A) Paired end reads from both Acid-Adapted (AA) and Non-Acid-Adapted (NA) MCF7 cell-lines were processed and transformed to high quality trimmed

reads then aligned to a reference genome to achieve count reads which have been used for differential expression analysis to explore Differential Expressed Genes (DEGs). Based on the STRING database an experimentally validated Gene Regulatory Network (confidence = 0.7) was constructed from DEGs. Network analysis was based on a scheme to explore and rank the network motifs with respect to EMT phenotype. **B)** Motif ranking has been performed using a multi objective weighting function and different network topological and biological parameters for nodes. Using weighting function leads to Pareto set of motifs which has been used to explore top 10 network motifs. These motifs were considered for further experimental validation.

supplementary figure 2.

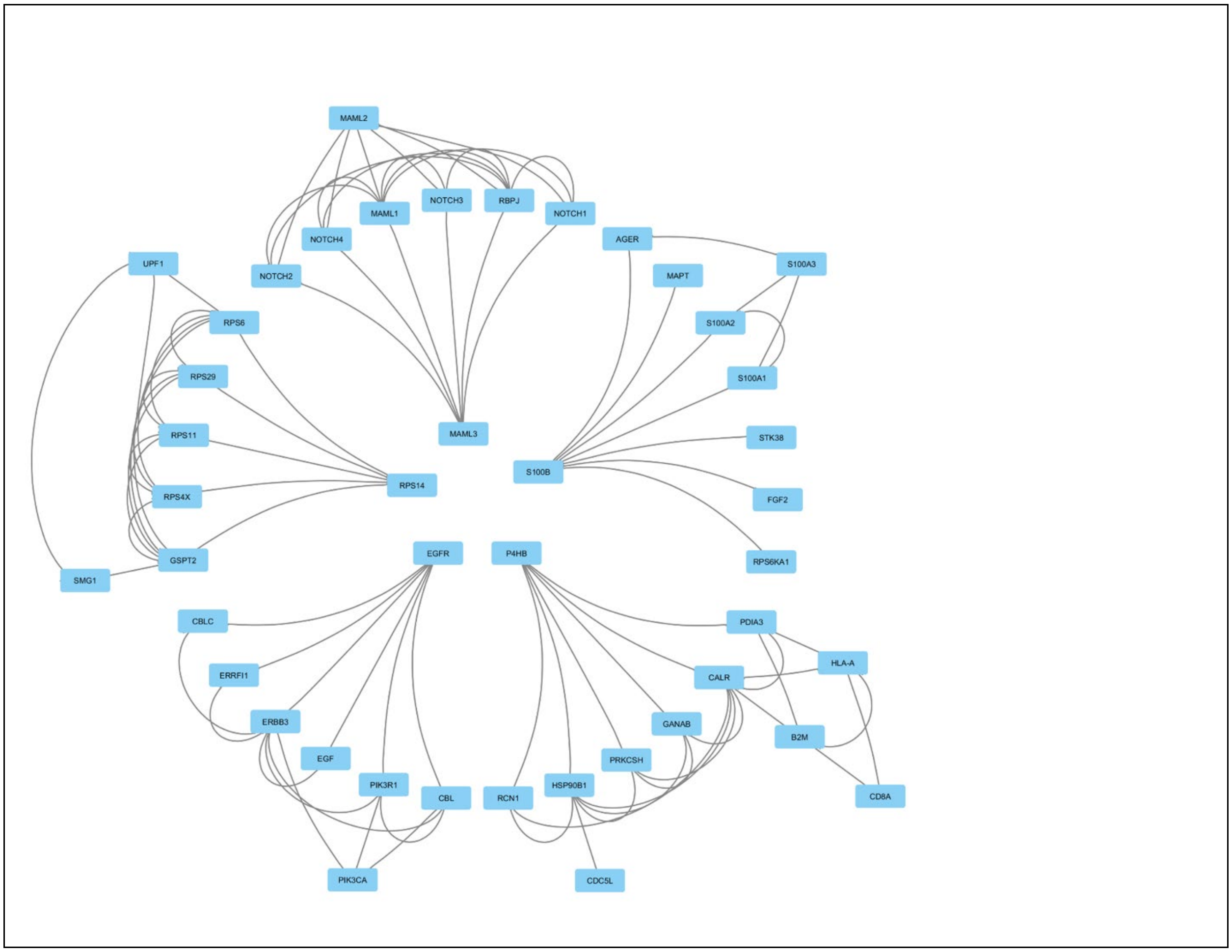

Sup figure 2: Interaction map of our motif packs, obtained from STRING database illustrating the first shell of interactions for each motif pack.

Supplementary Figure 3:

A)

2518

2375

323 2028 466

2518
Normal pH (light)
Low pH (heavy)

2375
normal pH (heavy)
Low pH (light)

2841
Combined Replicates
MaxQuant ID Criteria: PEP score ≤ 0.05
Maximum Intensity > 0

B)

≥ 1.5 stdev up in Low pH		≥ 2 stdev up in Low pH	
77	66	36	32
Low pH (light)	Low pH (heavy)	Low pH (light)	Low pH (heavy)
**≥ 1.5 stdev higher in Normal pH**		**≥ 2 stdev up in Normal pH**	
38	59	17	30
Normal pH (heavy)	Normal pH (light)	Normal pH (heavy)	Normal pH (light)

C)

Log2 Ratio H(Normal ph)/L(Low pH)

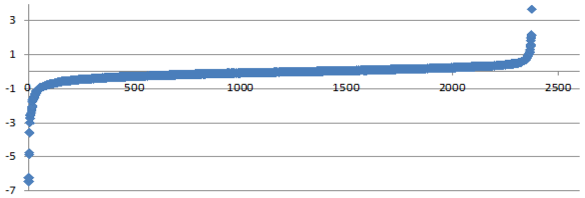

Average of log2 values = -0.081
(0.95-fold)

Stdev of log2 values = 0.4653

Median of log2 values = -0.0347
(0.98-fold)

Log2 Ratio H(Low ph)/L(Normal pH)

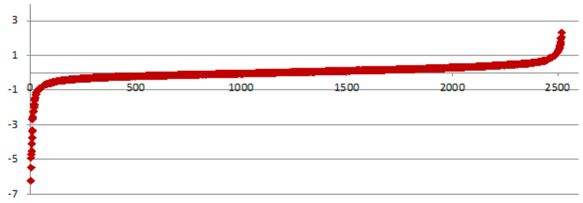

Average of log2 values = 0.024
(1.02-fold)

Stdev of log2 values = 0.4719

Median of log2 values = 0.0283
(1.02-fold)

Sup figure 3. SILAC proteomics analysis of DCIS breast cancer cells. A) Number of unique proteins in acid adapted and non-adapted cancer cells labeled with heavy and light isotopes. **B)** Unique proteins in each flipping experiment group with stdev cut off 1.5 and 2. **C)** Log 2 ratio showing the fold change of all the proteins detected in our SILAC proteomics experiment in both filliping experiments.

Supplementary Figure 4:

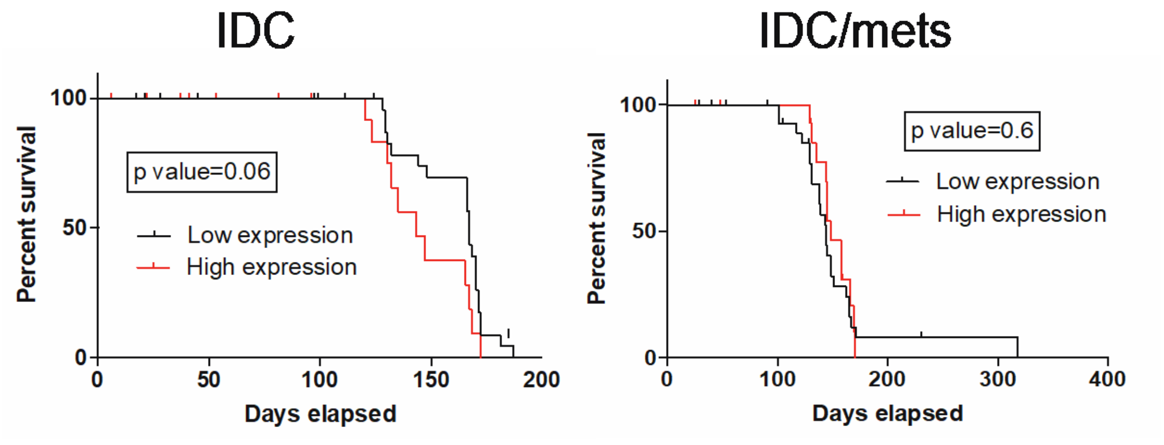

Sup figure 4: Survival analysis of patients with high and low expression of S100A6 in different stages from IDC, and IDC with local metastasis respectively.
